# Establishment and Characteristics of the Spermatogonial Stem Cell Line from the Yellow River Carp (*Cyprinus carpio haematopterus*)

**DOI:** 10.3390/biology14050536

**Published:** 2025-05-12

**Authors:** Huijie Zhou, Tianqi Liu, Tan Zhang, Zhipeng Sun, Huan Xu, Tingting Zhang, Yashan Yin, Na Li, Ting Yan, Youyi Kuang

**Affiliations:** 1 National and Local Joint Engineering Laboratory for Freshwater Fish Breeding, No. 232, Hesong Street, Daoli District, Harbin 150070, China; huij_zhou@163.com (H.Z.); liutianqi@hrfri.ac.cn (T.L.); tan_zhg@sina.com (T.Z.); sunzhipeng@hrfri.ac.cn (Z.S.); xuhuan@hrfri.ac.cn (H.X.); zhangtingting@hrfri.ac.cn (T.Z.); yinyashan1@163.com (Y.Y.); lina01251@163.com (N.L.); 2 Key Laboratory of Freshwater Aquatic Biotechnology and Breeding, Ministry of Agriculture and Rural Affairs, No. 232, Hesong Street, Daoli District, Harbin 150070, China; 3 Heilongjiang River Fisheries Research Institute of Chinese Academy of Fishery Sciences, No. 232, Hesong Street, Daoli District, Harbin 150070, China; 4 College of Fisheries, Tianjin Agricultural University, No. 22, Jinjing Road, Xiqing District, Tianjin 300384, China; 5 College of Fisheries and Life Sciences, Dalian Ocean University, No. 52, Heishijiao Street, Shahekou District, Dalian 116023, China

**Keywords:** *Cyprinus carpio haematopterus*, gonad stem cell culture, spermatogenesis, in vitro differentiation, transplantation

## Abstract

To address the growing consumer demands for improved fish meat quality, desirable morphological traits, and sustainable production practices, researchers have intensified efforts in the selective breeding and genetic improvement of carp (*Cyprinus carpio*) varieties. However, traditional breeding methods are often time-consuming and inefficient, which poses challenges to the sustainable development of the carp aquaculture industry. The establishment of germ stem cell lines offers a crucial tool for the study of germ cells, genetic improvement, and species conservation. In this study, we successfully established a spermatogonial stem cell line (YRSSCs) from Yellow River carp (*Cyprinus carpio haematopterus*) that can be cultured in vitro for the long term. We optimized the culture conditions to maintain their self-renewal and differentiation capabilities. The results demonstrated that YRSSCs have a diploid karyotype and can stably proliferate for over a year in L-15 medium supplemented with 5 mmol/L HEPES, 50 μmol/L β-mercaptoethanol, 15% FBS, 2 ng/mL bFGF, 2 ng/mL LIF, 1% carp serum, 800 IU/mL penicillin, 0.8 mg/mL streptomycin, 2 μg/mL amphotericin B, 1% zebrafish embryo extract, and 1% glutamine at 30 °C in the absence of CO_2_. The cells exhibited a typical germ stem cell gene expression profile, with strong expression of the *vasa*, *plzf-a*, and *Oct4-a* genes. Additionally, this study found that YRSSCs possess the ability to differentiate in vitro and functionally colonize in vivo within recipient bodies. This research explored the establishment of YRSSCs and their differentiation potential both in vitro and in vivo, providing a novel strategy for the genetic improvement of aquaculture fish species through germ stem cell-based gene editing and transplantation technologies.

## Simple Summary

Germ cell transplantation has been successfully performed in cyprinid fish; a stable long-term in vitro culture system for carp spermatogonial stem cells (SSCs) has not yet been established. This study developed an effective culture method for Yellow River carp (*Cyprinus carpio haematopterus*) SSCs (YRSSCs), maintaining their proliferation and stemness for over one year in optimized medium conditions. The cultured YRSSCs showed stable diploid maintenance and demonstrated both in vitro differentiation potential and in vivo colonization ability after transplantation. This work provides a reliable in vitro culture system for carp SSCs, creating new opportunities for genetic breeding applications in aquaculture.

## 1. Introduction

Fish germ stem cells are a highly specific and undifferentiated group of pluripotent cells located in the adult gonads, encompassing both male and female germ cells [1]. These cells originate from primordial germ cells (PGCs) during early embryonic development [2]. Germ stem cells (GSCs) have the ability to self-renew and differentiate into sperm or eggs and play a vital role in reproduction and serve as key transmitters of genetic information [3]. Currently, germ cells from various fish species, including zebrafish (*Danio rerio*) [4], medaka (*Oryzias latipes*) [5], rainbow trout (*Oncorhynchus mykiss*) [6], tilapia (*Opsariichthys bidens*) [7], and Japanese grenadier anchovy (*Coilia nasus*) [8], have been successfully isolated and cultured in vitro. The success of germ cell culture relies on efficient cell separation and stable in vitro conditions. Thus, specific separation and culture techniques need further development and optimization for most aquatic species [9].

The Yellow River carp (*Cyprinus carpio haematopterus*) is an economically significant freshwater fish native to the Yellow River basin in China [10]. It is renowned for its golden scales, red tail, elongated fusiform body, and delectable flesh [11,12]. To meet the growing consumer demand, numerous scientists have dedicated their efforts to the refined breeding and selection of carp strains [13]. However, traditional breeding methods are often time-consuming and inefficient, posing challenges to the sustainable development of the carp aquaculture industry [14]. With advancements in molecular biotechnology, genetic manipulation can now be performed directly at the cellular level. By introducing specific desirable genes into germ stem cells and employing techniques such as in vitro germ cell culture, germ cell transplantation, and gene editing, it is possible to rapidly develop new varieties with specialized traits. This approach significantly shortens the breeding cycle and enhances breeding efficiency [15,16,17]. The cell culture technology for cyprinid fish has seen rapid advancements. In zebrafish, PGCs and spermatogonial stem cells (SSCs) have been successfully cultured [18,19,20]. For *O. bidens*, long-term stable in vitro culture of SSCs has been established, and their differentiation has been induced 7. Using isolated SSCs from rare minnow (*Gobiocypris rarus*), gene editing has been performed, and the edited SSCs were transplanted into zebrafish recipients, producing genome-edited sperm derived from rare minnow [21].

While successful isolation and transplantation of mirror carp (*Cyprinus carpio*) germ cells into goldfish (*Carassius auratus*) resulting in donor-derived gametes has been demonstrated [22], no studies have reported long-term in vitro culture and differentiation of carp SSCs. This study was conducted to establish a reliable in vitro culture system for Yellow River carp SSCs (YRSSCs) capable of long-term maintenance and multilineage differentiation, thereby creating a technological foundation for generating improved aquaculture germplasm through germ cell-mediated genetic engineering.

## 2. Material and Methods

### 2.1. Animal Ethics

This study adhered to the Guidelines for the Management and Use of Laboratory Animals established by the Chinese Association for Laboratory Animal Sciences (Document No. 2011-2). All experimental protocols were approved by the Animal Management and Use Committee of the Heilongjiang Fisheries Research Institute, Chinese Academy of Fishery Sciences (Approval Code: 20230415-002).

### 2.2. PCR-Based Sex Identification

In the early developmental stages of fish, the gonads are not fully differentiated, resulting in a higher proportion of SSCs. Due to the undifferentiated state of the gonads in 10-month-old Yellow River carp, it is not possible to distinguish the sex of the fish by morphology. In this study, we utilized sex-specific primers (refer to Supplementary Table S1) developed from male and female genomes to identify candidate fish for SSC culture in vivo, as previously demonstrated in similar studies.

A small piece of the caudal fin ray of Yellow River carp was sampled, and genomic DNA was extracted using the Universal Genomic DNA Kit (CWBIO, Taizhou, China). The PCR amplification protocol was as follows: 95 °C for 5 min; 35 cycles of 95 °C for 30 s, 60 °C for 30 s, and 72 °C for 30 s; followed by a final extension at 72 °C for 5 min. The PCR products were visualized on a 1.5% agarose gel.

### 2.3. Isolation of SSCs

**Figure 1:**
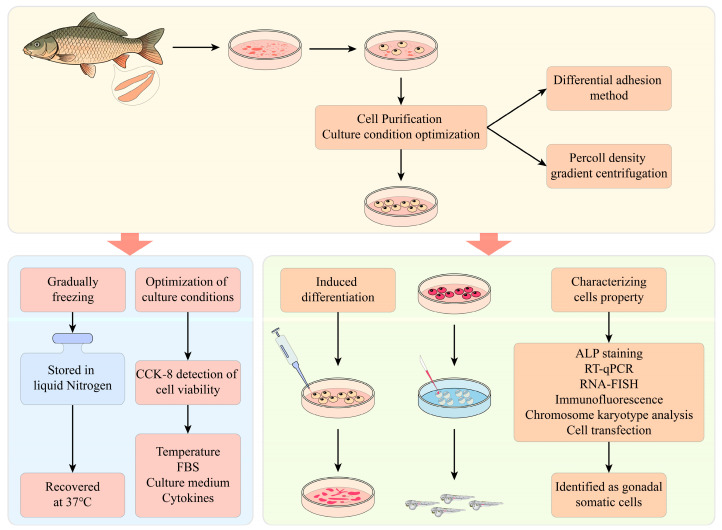
Flowchart of experiments. SSCs were isolated using tissue explant culture, followed by subculture to establish the cell line. Cell purification was achieved through differential plating and Percoll density gradient centrifugation. Cell viability was assessed by CCK-8 assay to optimize in vitro culture conditions (including temperature, FBS concentration, basal medium, and growth factors). Cryopreserved and thawed YRSSCs maintained stable viability and genetic characteristics. YRSSCs were characterized using ALP staining, RT-PCR, RNA-FISH, immunofluorescence, karyotype analysis, and transfection assays, confirming their spermatogonial stem cell identity. Pluripotency was further validated through in vitro differentiation and cell transplantation.

Yellow River carp at 10 months with an average body length of 116.87 mm and weight of 70.60 g were selected from the Hulan Experimental Station of the Heilongjiang Fisheries Research Institute, Chinese Academy of Fishery Sciences. Fish were anesthetized with MS-222 (MCE, Shanghai, China), disinfected with 0.1% potassium permanganate solution, and treated with 75% ethanol before their testes were dissected. The dissected testes were washed three times in PBS containing 8% penicillin-streptomycin-amphotericin B to remove other tissues. The testicular tissue was soaked in L-15 basal medium [Leibovitz L-15 medium (L-15, Gibco, Grand Island, NY, USA), 5 mM HEPES (Biosahrp, Hefei, China), 50 μmol/L β-mercaptoethanol (MCE, Shanghai, China)] for 20 min, then minced into 1 mm³ pieces in 200 μL carp serum. The tissue fragments were evenly spread onto culture dishes pre-treated with 1% gelatin and incubated at 30 °C without CO_2_ for 4 h. Then the cell culture medium was replaced with 2 mL of L-15 complete medium (L-15 basal medium, 15% FBS, 2 ng/mL bFGF, 2 ng/mL LIF, 1% carp serum, 800 IU/mL penicillin, 0.8 mg/mL streptomycin, 2 μg/mL amphotericin B, 1% zebrafish embryo extract, 1% glutamine) (Gibco, Grand Island, NY, USA). After 24 h, an additional 2 mL of L-15 complete medium was added, and the medium was subsequently replaced every two days. The isolated SSCs were used for subsequent experiments (Figure 1). In this study, two additional primary culture methods were employed for screening the most suitable primary culture method for YRSSCs. In one method, the testes were cut into 1 mm³ pieces and digested for 2 h in 1 mL of 0.25% trypsin and 50 μL of carp serum. After termination by adding 2 mL of L-15 complete medium, the cell suspension was filtered through a 100 μm sterile cell strainer before culturing. The chelating agent dissociation method followed the same procedure as the trypsin digestion method, except that 1 mM EDTA was added to the 0.25% trypsin solution.

### 2.4. Cell Passaging and Purification

Cell passaging was conducted according to the method used for the turbot brain cell line [23]. After reaching the 10th passage, YRSSCs were passaged approximately every 4–5 days. Purification of YRSSCs was achieved using the differential adhesion method and Percoll density gradient centrifugation to remove fibroblasts.

During passages 4 to 15, YRSSCs were purified using differential plating. After digestion with 0.25% trypsin-EDTA (Gibco, Grand Island, NY, USA), the cell suspension was transferred to culture dishes and incubated for 40 min to allow some fibroblasts to adhere. The non-adherent cells were then transferred to new culture dishes for continued cultivation, completing one cycle of differential plating.

In addition to the differential plating method, we also carried out Percoll density gradient centrifugation for purifying SSCs. An isotonic solution was prepared by mixing 8.5% NaCl with Percoll stock solution in specific proportions to create gradients of 10%, 20%, 30%, 40%, and 50%. These solutions were sequentially layered into a 15 mL centrifuge tube, with the highest density at the bottom. The cell suspension was gently added to the centrifuge tube. After centrifugation at 1400 rpm for 25 min, the cell bands formed at different gradients were carefully transferred to new tubes, washed twice with L-15 complete medium, and then seeded into new culture dishes.

### 2.5. Cell Cryopreservation and Recovery

When cell confluency reached 80% to 90%, the cells were washed three times with PBS, digested with 0.25% trypsin-EDTA for 5 min, and the digestion was terminated with L-15 complete medium. The cells were then centrifuged at 1200 rpm for 5 min, the supernatant was discarded, and fresh cryopreservation solution was added before transferring to dedicated cryovials. The cells were initially stored overnight at −80 °C, then transferred to a liquid nitrogen tank for long-term storage. This study explored the optimal cryopreservation conditions for the cells by setting up two groups of cryopreservation solutions: Solution 1 with a mixture of L-15 complete medium, FBS, and DMSO with a ratio of 7:2:1, or Solution 2 with a mixture of FBS and DMSO with a ratio of 10:1.

To resuscitate the frozen cells preserved in liquid nitrogen, the frozen tube was immediately transferred to a 37 °C water bath. Once the medium had melted, 10 mL of L-15 complete medium was added, and the tube was centrifuged at 1200 rpm for 5 min. Subsequently, the cells were resuspended in L-15 medium and cultured at 30 °C without CO_2_.

### 2.6. Optimization of Culture Conditions

To determine the optimal culture conditions for YRSSCs, the study evaluated cell proliferation under varying conditions of temperature (26 °C, 28 °C, and 30 °C), serum concentrations (5%, 10%, 15%, and 20%), basal media (L-15, DMEM, DMEM-F12, and M199), and the absence of different growth factors (bFGF, LIF, carp serum, zebrafish embryo extract, and glutamine). Each group had only one variable, with all other conditions kept constant.

Cells were seeded at a density of 5000 cells/well in a 96-well plate. After seeding, the medium was changed every two days, and 10 μL of CCK-8 reagent (Invigentech, Carlsbad, CA, USA) was added at the same time each day. After incubating for 2 h and 30 min, the optical density (OD) value at 450 nm was measured, continuing until day 6.

### 2.7. Karyotype Analysis

Karyotype analysis was performed with YRSSCs at passage 30. A final concentration of 0.4 μg/mL colchicine (Beyotime, Nantong, China) was added to a T25 cell culture flask, and the culture was continued for 14 h. After digestion with 0.25% trypsin-EDTA, cells were collected. The cells were treated with hypotonic 0.075 mol/L KCl solution at room temperature for 40 min, followed by centrifugation and resuspension in pre-cooled Carnoy’s fixative (formaldehyde: glacial acetic acid = 3:1) for pre-fixation of 5 min. After discarding the supernatant, cells were resuspended in Carnoy’s fixative and fixed at room temperature for 20 min, repeating the process three times. The fixed cell suspension was dropped onto pre-cooled slides, air-dried, and then stained with 10% Giemsa stain (Solarbio, Beijing, China). The stain was gently washed off with distilled water. Under the microscope, 100 metaphase chromosomes were observed, the number of chromosomes was counted, and photographs were taken.

### 2.8. Alkaline Phosphatase Staining

The 30th passage of YRSSCs was seeded into a 24-well plate and was cultured until the cell confluency reached 80% to 90%, followed by a 3–5 min wash with PBS. The cells were fixed with 95% alcohol for 30 min and washed again with PBS for 3–5 min. Staining was performed using a BCIP/NBT Alkaline Phosphatase Color Development Kit (Beyotime, Nantong, China). The staining reaction was terminated by washing with distilled water. Observations and photographs were taken under a microscope.

### 2.9. Expression of Germ Cell Marker Genes

The 15th passage of YRSSCs was seeded into a 6-well plate and cultured until reaching 90% confluency. Total RNA was extracted using TRIzol (Invitrogen, Carlsbad, CA, USA). Genomic DNA removal and reverse transcription were conducted following the PrimeScript™ RT reagent Kit with gDNA Eraser protocol (Takara, Otsu, Japan), with the resulting cDNA being used as a template (the cDNA was diluted 10-fold before use) for PCR amplification detection. The PCR primer sequences for *vasa*, *nanos1*, *nanos3*, *foxl2a*, *klf4*–2, and *β*-*actin* are listed in Supplementary Table S2, *β-actin* was used for the internal reference gene.

### 2.10. RNA-FISH

The RNA-FISH probes for genes *vasa*, *dmrt1-a*, *plzf-a*, and *Oct4-a* (*vasa-a*: ENSCCRT00000142012; *vasa-b*: ENSCCRT00000178331; *dmrt1-a*: ENSCCRG00000032706; *plzf-a*: ENSCCRG00000053566; *Oct4-a*: ENSCCRG00000059812) were designed according to the method described by Kishi et al. [24]. The probe sequences are shown in Supplementary Tables S3 and S4.

Testes from 10-month-old male Yellow River carp were fixed overnight at 4 °C in 4% PFA, dehydrated through a sucrose gradient, embedded in OCT (Biosahrp, Hefei, China), and sectioned into 5 μm-thick slices using a cryostat (CM 1950, Leica, Wetzlar, Germany). A single-cell suspension of the 20th passage YRSSCs was transferred to the 12-well plate with smears. Once cell confluency reached 70% to 80%, cells were fixed with 4% paraformaldehyde. The cells were permeabilized with PBS and 0.5% Triton X-100 at room temperature for 10 min. SABER-FISH was performed according to the protocol of Kishi et al. [24]. A 1 μg/mL DAPI (Biosharp, Shanghai, China) staining solution was evenly dropped onto the cell samples and stained at room temperature for 5 min, followed by washing with PBS. After the slides were air-dried, observations were made using a fluorescence inverted microscope.

### 2.11. Flurorescence in Situ Hybridization of Vasa

The preparation of cell smears is the same as RNA-FISH. Cells were fixed with 4% paraformaldehyde and permeabilized with PBS + 0.5% Triton X-100 at room temperature. Blocking was performed with 5% skim milk at 28 °C for 30 min. The primary antibody against *vasa* antibody (1:200, PA5-30749, Invitrogen, Carlsbad, CA, USA) was incubated overnight at 4 °C. After washing with TBST, a fluorescent Goat Anti-Rabbit IgG H&L antibody (1:100, bs0295GFITC, Bioss, Beijing, China) was added and incubated at 28 °C for 1 h in the dark. After washing, the nuclei were stained with 1 μg/mL DAPI for 5 min. Anti-fade mounting medium was added to the coverslips, and the slides were sealed. Photographs were taken using a fluorescence microscope.

### 2.12. Analysis of the YRSSCs’ Ability to Express Exogenous Genes

To evaluate the ability of YRSSCs to express exogenous genes, the pEGFP-N1 plasmid expressing green fluorescent protein was used to assess the transfection capability of the cells, prepared according to the protocol of Lipofectamine 3000 reagent (Invitrogen, Carlsbad, CA, USA). YRSSCs were seeded in a 12-well plate and cultured for 16–20 h. The medium was then replaced with Opti-MEM (Gibco, Grand Island, NY, USA) for further culturing. A mixture of 1 μg pEGFP-N1 plasmid and Lipofectamine 3000 reagent was incubated at room temperature for 15 min and then added dropwise to the surface of the culture medium. Cells were observed under a fluorescence microscope 12 h post-transfection. To improve transfection efficiency, passage 30 YRSSCs were transfected according to the ZETA transfection kit (3443S, ZETA Life Biotechnology Co., Ltd., Shanghai, China) protocol. The ZETA reagent and plasmid DNA were mixed at a 1:1 ratio and added to the cell culture medium. After 24 h of incubation, the medium was replaced, and transfection efficiency was assessed 48–72 h post-transfection.

YRSSCs were seeded in a 96-well plate at a density of 1 × 10^4^ cells/well, with 100 μL of medium per well. On the second day, when the cell density reached 30–50%, the medium was removed. The HBAD-EGFP (HANBIO, Shanghai, China) virus at a concentration of 1 × 10^9^ PFU/mL was diluted according to the MOI value (MOI = (virus concentration × volume)/number of cells), with MOI values set at 200 and 500. The L-15 complete medium was supplemented to a final volume of 50 μL, and after 4 h of infection, an additional 100 μL of L-15 complete medium was added. Sixteen hours post-infection, the virus-containing medium was aspirated and replaced with fresh L-15 complete medium. Forty-eight hours post-infection, photographs were taken for observation.

### 2.13. In Vitro-Induced Differentiation

The 25th passage YRSSCs were seeded into a 6-well plate at a density of 2 × 10^5^ cells per well and cultured for 24 h. The cells were then induced to differentiate by adding 10 μM retinoic acid (MCE, Shanghai, China). The differentiation process was continued for 14 days, and the differentiation status of the cells was observed under a microscope.

### 2.14. Cell Transplantation

The 25th passage YRSSCs were stained using the PKH26 dye (Solarbio, Beijing, China) for subsequent cell transplantation. After treatment with dnd1-MO (50 μM, Gene Tools, LLC, Philomath, OR, USA), the blastula embryos of zebrafish were used as recipients for cell transplantation; approximately 100 to 300 YRSSCs were injected into the embryos. The embryos were cultured at 28.5 °C, with dead eggs removed and the water changed twice daily. The embryos were monitored regularly, and the fluorescently labeled germ stem cells were tracked under a fluorescence microscope.

### 2.15. Statistical Analysis

Statistical analysis was performed by one-way variance analysis and Student’s *t*-test. A probability level less than 0.05 (*p* < 0.05) was considered statistically significant. All statistics were implemented using GraphPad Prism 9.5.0 (USA).

## 3. Results

### 3.1. Sex Identification of Yellow River Carp

**Figure 2:**
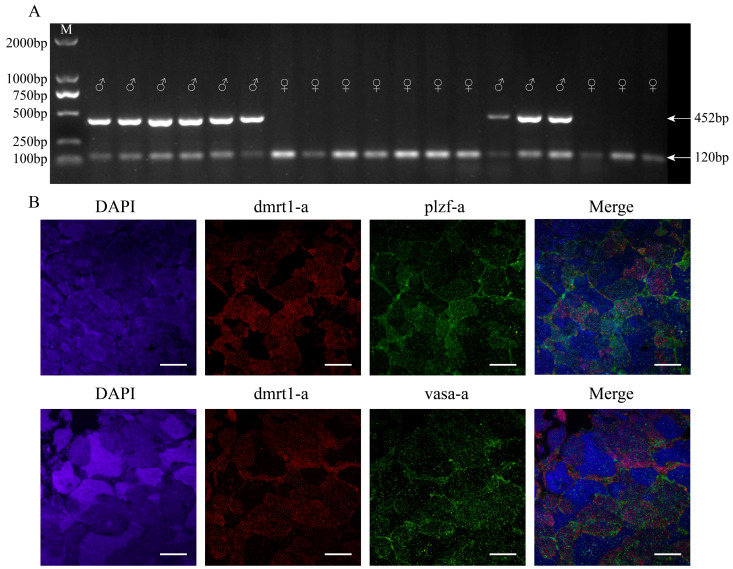
Results of male and female identification of Yellow River carp (*Cyprinus carpio haematopterus*). (**A**) M: DL2000; The arrow points to the target fragment; ♂ is for male fish; ♀ is for female fish. (**B**) RNA-FISH analysis demonstrated co-localization of *dmrt1-a* with both *plzf-a* and *vasa-a* transcripts in 10-month-old Yellow River carp testes. Scale bars 100 μm.

The PCR-based sex identification results demonstrated that female Yellow River carp exhibited a single 120 bp band, while males displayed two distinct bands of 120 bp and 452 bp, respectively (Figure 2A). Following this genotyping, testicular tissues were dissected from the identified male specimens. Further analysis using RNA-FISH technology with specific markers (the somatic cell marker gene: *dmrt1-a*, the germ cell marker gene: *vasa*, and the SSC marker gene: *plzf-a*) revealed co-localization patterns of *dmrt1-a*/*vasa-a* and *dmrt1-a*/*plzf-a.* These results demonstrated the presence of abundant SSCs in the testes of 10-month-old Yellow River carp, providing an ideal cellular source for subsequent SSC isolation and culture (Figure 2B).

### 3.2. Establishment of a Spermatogonial Stem Cell Line

**Figure 3:**
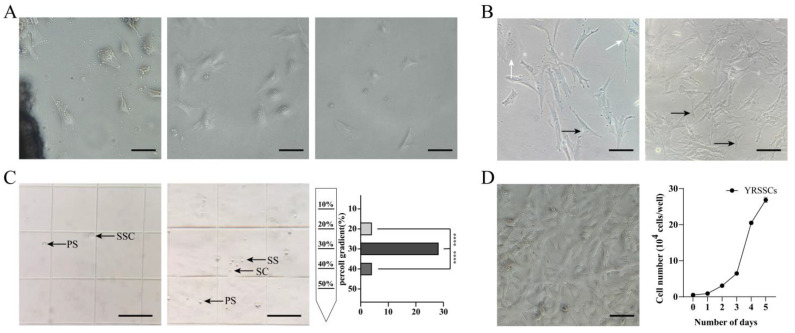
Isolation, purification, and establishment of YRSSCs. (**A**) Primary isolation of SSCs using three distinct methods: tissue explant culture, trypsin digestion, and chelating agent dissociation. (**B**) Purification of YRSSCs by differential plating. Cell morphology before (**left**) and after (**right**) purification is shown. Black arrows indicate SSCs; white arrows denote fibroblasts. (**C**) Further purification of YRSSCs by Percoll density gradient centrifugation. Cell layers at 30% and 40% Percoll concentrations are displayed, along with the proportion of SSCs at different densities. SSC: spermatogonial stem cell; PS: primary spermatocyte; SS: secondary spermatocyte; SC: spermatid. ****, *p* < 0.0001. (**D**) Morphology and growth curve of passage-60 YRSSCs after purification and stable expansion using tissue explant culture. Scale bar: 30 μm.

SSCs were isolated from the testes of Yellow River carp using tissue adherence, trypsin digestion, and chelating agent dissociation methods. Utilizing the tissue adherence method to isolate testes from 10-month-old Yellow River carp, elongated, triangular, and irregular polygonal cells were observed to migrate out from the tissue fragments and extend outward on the third day. The trypsin digestion method yielded a large number of single cells suspended in the medium, with triangular and irregular polygonal cells observed adhering to the surface by the seventh day of isolation; however, many white suspended cells remained non-adherent. Using the chelating agent dissociation method, where 1 mM EDTA was added to the 0.25% trypsin digestion, single suspended cells were also isolated. By the fifth day, only a few cells isolated from the testes adhered to the surface, displaying irregular polygonal shapes (Figure 3A). Through comparative analysis, the tissue adhesion method demonstrated significant advantages in the isolation and culture of YRSSCs. The cells exhibited good growth conditions and uniform morphology. They have now been stably passaged to the 60th generation, with a confluency of 100% achieved within 4–5 days after seeding (Figure 3D). Therefore, we recommend adopting the tissue adhesion method for the isolation and culture of SSC cell lines in future studies.

In this study, differential adhesion and Percoll density gradient centrifugation methods were used to purify YRSSCs. Under the microscope, a large number of fibroblasts were observed in the 1st to 3rd passages of YRSSCs. Based on the characteristic that fibroblasts adhere more easily than germ stem cells, differential adhesion purification was able to remove some of the fibroblasts (Figure 3B). The SSCs continued to proliferate normally after the partial removal of fibroblasts. Concurrently, SSCs were purified using the Percoll density gradient method with five gradients: 10%, 20%, 30%, 40%, and 50%. The results showed that the cell layer at 30% Percoll concentration contained SSCs and primary spermatocytes, while the cell layer at 40% Percoll concentration contained a variety of cell types, including primary spermatocytes, secondary spermatocytes, spermatids, and sperm. Analysis revealed that the cell layer at 30% Percoll concentration had a significantly higher proportion of germ stem cells compared to the 20% and 40% layers (*p* < 0.01) (Figure 3C). SSCs isolated from the testes of Yellow River carp gradually became morphologically homogeneous after differential adhesion and Percoll density gradient centrifugation. The purified SSCs reached a purity of 70.16% by the 14th passage.

### 3.3. Optimization of Cell Culture and Cryopreservation Conditions

**Figure 4:**
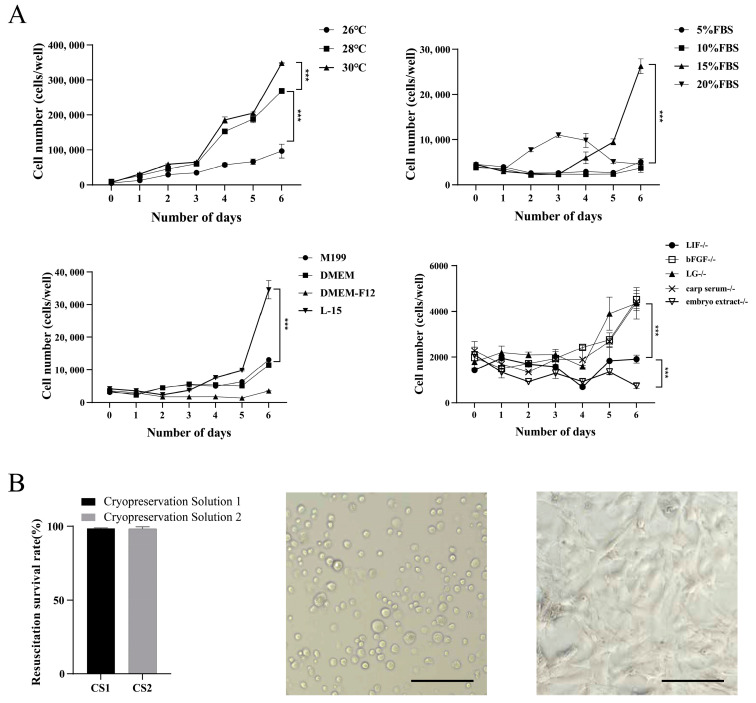
Optimization of Culture and Cryopreservation Conditions for YRSSCs. (**A**) The proliferation kinetics of carp SSCs were systematically evaluated through 6-day culture experiments examining: temperature effects (26 °C, 28 °C, 30 °C); basal media formulations (M199, DMEM, DMEM/F12, Leibovitz’s L-15); FBS concentration gradients (5%, 10%, 15%, 20% in L-15 complete medium); and growth factor requirements via component-deprived conditions (L-15 medium lacking LIF, bFGF, laminin gel, carp serum, or embryonic extract). Graphical representation of cell proliferation showing: -Vertical axis: Cell number-Horizontal axis: Culture time (days) Data are mean ± SEM (*n* = 3). (**B**) Cell recovery viability rates were compared between Cryopreservation Solution 1 and Solution 2 (*n* = 3 replicates). Post-thaw analysis revealed typical morphology prior to adhesion and normal cellular status at 2 days post-adhesion. Scale bars 100 μm.

To explore the optimal growth conditions for YRSSCs, the 12th passage of stably passaged YRSSCs was subjected to cell proliferation assays under different temperatures, media, serum concentrations, and growth factor deficiencies over a period of 0–6 days. The results showed that YRSSCs could stably proliferate at temperatures of 26 °C, 28 °C, and 30 °C. However, by day 6, the count of cells at 30 °C was significantly higher than that at 26 °C and 28 °C (*p* < 0.01), and the cell count at 28 °C was significantly higher than at 26 °C (*p* < 0.01). When cells were cultured in M199 and DMEM media, YRSSCs exhibited minimal proliferation, and even growth inhibition was observed. In contrast, L-15 medium supported normal proliferation of YRSSCs, and by day 6, the count of cells in L-15 medium was significantly higher than that in M199, DMEM, and DMEM-F12 media (*p* < 0.01). Among different FBS concentration groups, YRSSCs proliferated normally only at 15% FBS, and by day 6, the count of cells was significantly higher than that at 5%, 10%, and 20% FBS concentrations (*p* < 0.01). In the growth factor deficiency groups, the absence of LIF, bFGF, LG, carp serum, and zebrafish embryo extract affected YRSSC proliferation. By day 6, the count of cells in media lacking zebrafish embryo extract and LIF was significantly lower than that in media lacking other growth factors (*p* < 0.01) (Figure 4A). This study determined that the optimal culture medium is L-15 supplemented with 15% FBS, along with LIF, bFGF, LG, carp serum, and zebrafish embryo extract. The optimal culture temperature is 30 °C.

YRSSCs were cryopreserved using two different cryopreservation solutions. After six months of storage, the cell recovery viability rates for Cryopreservation Solution 1 and Cryopreservation Solution 2 were 99.66% and 98.39%, respectively; no significant difference was observed between the two solutions. Following resuscitation, YRSSCs initially exhibited spherical morphology prior to attachment. After 48 h of culture, the cells demonstrated robust proliferation, achieving 90% confluence (Figure 4B).

### 3.4. Characterization of YRSSCs

**Figure 5:**
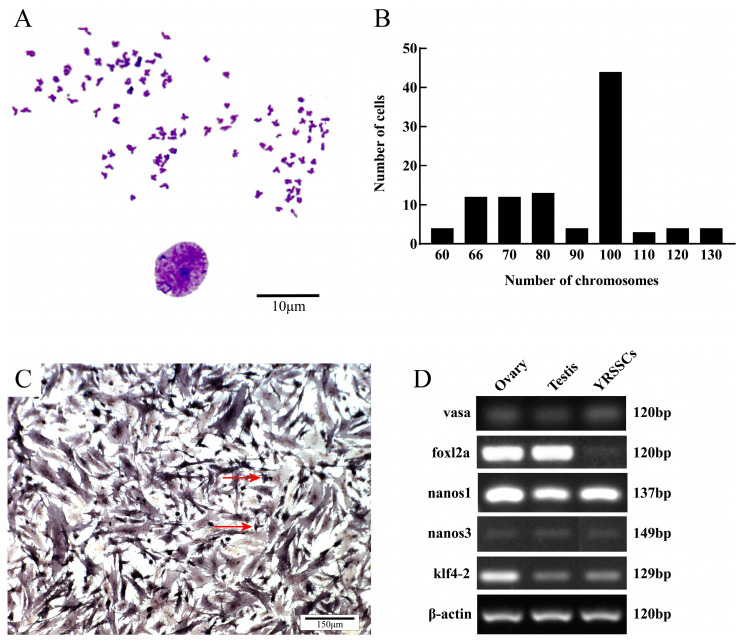
Karyotype Analysis, ALP Staining, and RT-PCR Analysis of YRSSCs. (**A**) Diploid metaphase of YRSSCs. Scale bar 10 μm. (**B**) Chromosome numbers were counted in colchicine-treated YRSSCs at passage 30. (**C**) ALP staining of YRSSCs. Scale bar 150 μm. ALP-positive cells are indicated with red arrowheads. (**D**) Semi-quantitative results of specific gene expression in gonadal tissue and YRSSCs, the original gel images were listed in Supplementary Figures S1–S5.

We characterized the YRSSCs by a series of experiments. Firstly, the chromosome analysis showed that 44% of the YRSSCs retained a diploid karyotype with 100 chromosomes by counting 100 well-spread and clearly defined metaphase cells (Figure 5A,B). Secondly, alkaline phosphatase was used as a phenotypic marker for germ stem cells. We performed ALP staining and found that YRSSCs were strongly positive for alkaline phosphatase staining (Figure 5C). Thirdly, the gene expression analysis revealed that the germ cell-specific marker genes *vasa*, *foxl2a*, *nanos1*, *nanos3*, and *klf4*-*2* were expressed in the SSCs (Figure 5D, Figures S1–S5), with *nanos1* and *klf4-2* showing significantly higher expression levels compared to testicular tissue.

**Figure 6:**
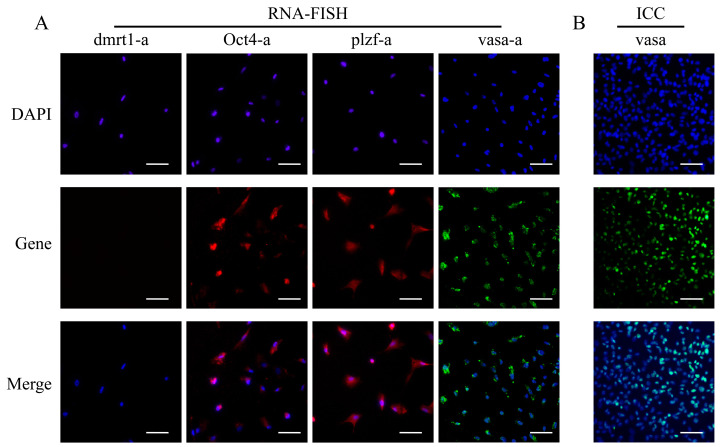
Analysis of *vasa* Gene Expression at the RNA and Protein Levels in YRSSCs and Evaluation of the Exogenous Gene Expression Capability in YRSSCs. (**A**) Expression of *dmrt1-a, Oct4-a*, *plzf-a*, and *vasa-a* genes at the RNA level in YRSSCs. (**B**) Expression of the *vasa* gene at the protein level in YRSSCs.

To assess the purification efficacy of SSCs, we conducted comprehensive analyses using RNA-FISH and immunofluorescence staining to examine germ cell markers (*vasa*), stemness markers (*plzf-a* and *Oct4-a*), and somatic cell markers (*dmrt1-a*). RNA-FISH analysis revealed robust co-expression of *vasa-a*, *plzf-a*, and *Oct4-a* transcripts in the cytoplasm of YRSSCs, while *dmrt1-a* expression was undetectable (Figure 6A). Quantitative evaluation demonstrated that 85.19% of cells exhibited strong co-expression of both *vasa-a* and *vasa-b* isoforms (Figure S6), indicating an 85.19% purity for passage 21 YRSSCs. This finding was further corroborated by immunofluorescence analysis, which showed positive *vasa* protein expression in virtually all cells examined (Figure 6B). These results collectively confirm the successful isolation and high purity of our SSC population.

**Figure 7:**
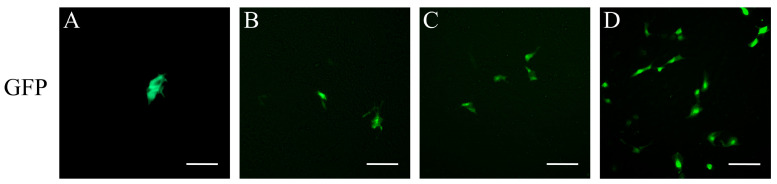
Transfection and infection efficiency analysis in YRSSCs. (**A**) GFP expression following pEGFP-N1 plasmid transfection in passage 15 cells. (**B**) Adenoviral transduction efficiency at MOI 200. (**C**) Adenoviral transduction efficiency at MOI 500. (**D**) GFP expression following pEGFP-N1 plasmid transfection in passage 30 cells. Scale bars: 50 μm.

Lipofection successfully introduced pEGFP-N1 green fluorescent protein into YRSSCs, and the expression of the EGFP gene was observed 12 h after transfection. Analysis revealed that the transfection efficiency of lipofection was low, at only 2.13% (Figure 7A). Additionally, the EGFP delivered with adenoviruses was also successfully transfected into YRSSCs, achieving a higher transfection efficiency compared to pEGFP-N1. At an MOI of 200, the efficiency reached 2.96% (Figure 7B), and at an MOI of 500, it reached 4.26% (Figure 7C). Following cell purification, replacement with ZETA liposomal transfection reagent, and optimization of transfection conditions, the transfection efficiency of passage 30 YRSSCs was enhanced to 24.39% (Figure 7D).

### 3.5. Induction of Differentiation In Vitro

**Figure 8:**
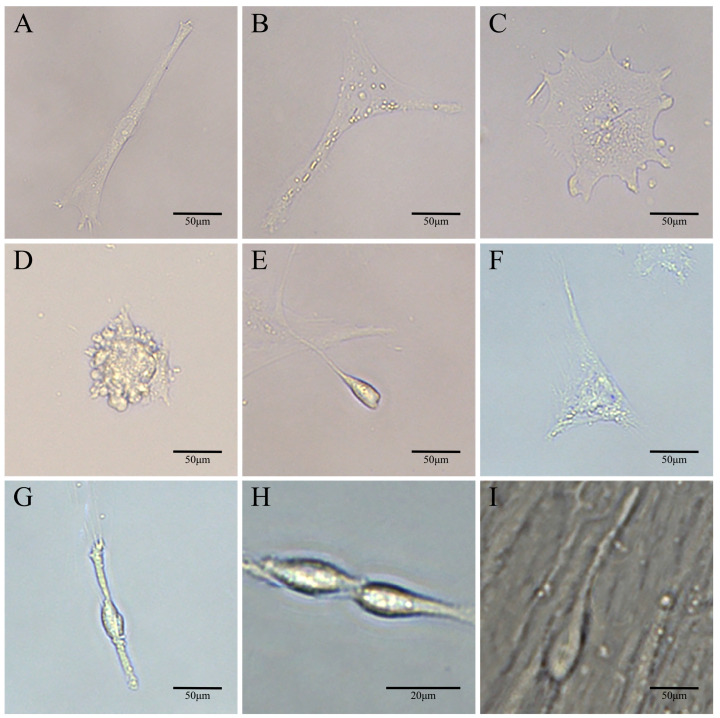
Induced differentiation of SSCs in vitro. (**A**) Nerve cells. (**B**) Astrocyte. (**C**) Epithelial cell. (**D**) Embryoid body. (**E**) Sperm head cells. (**F**) Astrocyte. (**G**) Nerve cell. (**H**) Sperm head-like cells. Scale bar 20 μm. (**I**) Sperm-like cell. Scale bar A-G, I 50 μm.

On the second day after being induced by 10 μM retinoic acid, a small number of neuronal cells, astrocytes, epithelial cells, embryoid bodies, and spermatid head-like cells could be observed (Figure 8A–E). On the seventh day after induction, a large number of astrocytes were observed, and the number of neuronal cells and spermatid head-like cells began to increase (Figure 8F–H). By the fourteenth day of induction, sperm-like cells were clearly observed on the surface of the cell layer (Figure 8I).

### 3.6. Pluripotency of YRSSCs In Vivo

**Figure 9:**
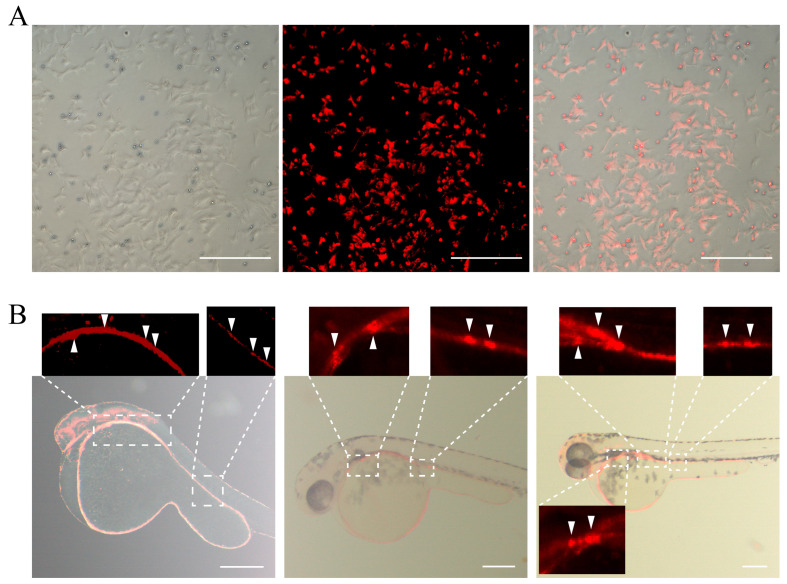
PKH26 Staining and Transplantation of SSCs. (**A**) PKH26 Staining: white light; PKH staining diagram; fusion diagram. (**B**) Spermatogonial stem cell colonization of chimeras at different stages of zebrafish receptor. The white arrows indicate the engraftment sites of YRSSCs. Scale bar 200 μm.

The cells stained with PKH26 (Figure 9A) were collected and transplanted into zebrafish blastula-stage embryos by microinjection. The results showed (Figure 9B) that cells with red fluorescence were observed to colonize in the reproductive-related regions and migrate towards the gonadal ridge.

**Figure 10:**
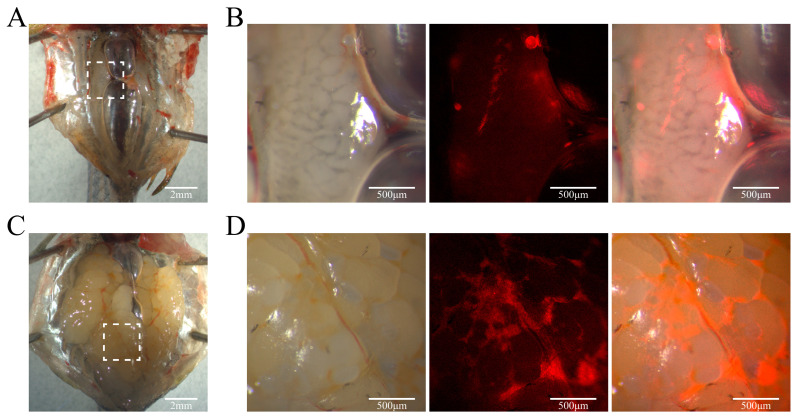
YRSSCs engraftment in the gonads of zebrafish chimeras. (**A**) Male zebrafish chimera at 9 months of age. Scale bar 2mm. (**B**) Enlarged view of the white box area in Figure A. Scale bar 500 μm. (**C**) Female zebrafish chimera at 9 months of age. Scale bar 2mm. (**D**) Enlarged view of the white box area in Figure C. Scale bar 500 μm.

When the chimeric zebrafish reached 9 months of age, red fluorescence was observed on the gonads. In the male chimeras, the testes developed normally, with red fluorescence displaying a punctate distribution on the surface of the testes, indicating that the transplanted germline stem cells successfully engrafted and may have contributed to testicular development (Figure 10A). In the female chimeras, the ovaries also developed normally, with red fluorescence showing clustered distribution within the ovaries, suggesting that YRSSCs may be involved in the ovarian development process (Figure 10B).

## 4. Discussion

### 4.1. Establishment of the YRSSCs

As the gonads develop, germ stem cells differentiate into various reproductive cells. Germ stem cells isolated from mature gonads not only have low purity but also contain somatic cells, such as fibroblasts and epithelial cells. In order to attain a purer germ stem cell line, our study employed differential adhesion and density gradient centrifugation, ultimately achieving a cell purity of 85.19% after 21 passages of YRSSCs. Previous studies have demonstrated that SSCs exhibit distinct density distribution patterns in Percoll gradients. In loach, approximately 60% of type A and early type B spermatogonia were found to concentrate within the 30–36% Percoll fraction [25]. Similarly, rare gudgeon SSCs showed preferential localization in the 35% gradient layer [21]. These findings are consistent with the result of this study, where the highest proportion and quantity of SSC were observed at the 30% Percoll density.

The optimization of in vitro culture systems for fish SSCs requires systematic evaluation of the synergistic effects among growth factors, culture temperature, and medium components [23]. The optimal culture temperature for warm-water freshwater fish has been reported to be between 25 and 30 °C, with L-15 medium demonstrating superior efficacy for fish cell line proliferation compared to other media formulations [26]. High concentrations of serum may inhibit cell growth, with FBS concentrations typically ranging from 5% to 20%, but should not exceed 20% [27]. This study determined that 30 °C represents the optimal culture temperature, with Leibovitz’s L-15 medium supplemented with 15% FBS providing the most favorable conditions. The growth factors that support cell proliferation include bFGF, LIF, carp serum, zebrafish embryo extracts, and glutamine. There are no fixed rules for supplementing growth factors; it is essential to investigate the required growth factors specifically for the fish species studied and for each organ involved in cell culture development [28]. Although the absence of bFGF in this study did not significantly affect cell proliferation, some studies have shown that bFGF can maintain the morphology of germ cells and, in conjunction with LIF, can significantly accelerate cell growth [29]. LIF, a multifunctional cytokine from the interleukin-6 (IL-6) family, mediates the self-renewal of pluripotent embryonic stem cells and inhibits differentiation [30]. Additionally, carp serum and zebrafish embryo extracts play a crucial role in supplementing the necessary nutrients for cell growth [8,31,32].

### 4.2. Characteristics of YRSSCs

Research indicates that *vasa* serves as a germ cell-specific marker gene, exhibiting dynamic expression patterns in the germ cells of various fish species and most tested organisms. This suggests its crucial role in germline development [33]. Oct4-a and plzf-a serve as pluripotency marker genes in fish, exhibiting characteristic expression patterns in embryonic stem cells, SSCs, and induced pluripotent stem cells (iPSCs) [34,35,36,37]. Dmrt1-a, a key regulator of sex determination in vertebrates, is specifically expressed in gonadal somatic cells and plays crucial roles in testis formation and male sexual development [38]. Molecular characterization of YRSSCs through RNA-FISH analysis revealed markedly elevated expression levels of *vasa-a*, *Oct4-a*, and *plzf-a* genes, while no detectable expression signal was observed for dmrt1-a. This distinctive gene expression profile exhibits high concordance with established molecular markers of fish germ stem cells, providing molecular evidence that YRSSCs possess characteristic properties of germline stem cells. Furthermore, RNA-FISH results showed that the expression level of the *vasa* gene in the cytoplasm was higher compared to that in the nucleus, aligning with the findings of Xu’s research [39].

Additionally, through the detection of the expression of germ cell marker genes, it was found that, besides the *vasa* gene, *foxl2a*, *nanos1*, *nanos3*, and *klf4* were also expressed in YRSSCs. The functionally conserved *nanos* gene family plays an important role in the maintenance of fish germ stem cells [40], with nanos1 being essential for maintaining the vitality of germ cells [41] and *nanos3* involved in germ cell function, serving as a marker for germ cells. GFP-nos1 3′UTR mRNA and GFP-nos3 3′ UTR mRNA allow for more stable expression of the GFP gene in germ cells and have been widely used in studies on the migration and localization of germ cells in zebrafish, goldfish, Acipenser sturio (Linnaeus, 1758), Thunnus thynnus (Linnaeus, 1758), and medaka embryos [42,43,44]. Studies have shown that mammalian *klf4* can regulate the self-renewal of embryonic stem cells, and in zebrafish, *klf4* can maintain the proliferation of epidermal stem cells by regulating the expression of p53 and cdkn1a/p21 [45,46]. In the study of stem cells in the flatworm (Bipalium kewense Moseley, 1878), the expression of *klf4* was detected in its PGCs, GSCs, and yolk precursor cells [47]. In this study, RT-PCR detected the expression of germ cell-specific genes *vasa, foxl2a, nanos1, nanos3, and klf4* in YRSSCs, proving that the established germ cells possess characteristics of germ cells, self-renewal, and differentiation capabilities. Among them, YRSSCs showed high levels of expression in *nanos1* and *klf4-2* genes, indicating the pluripotency and differentiation potential of these cells.

Compared with mammalian cell lines, PGCs of teleost fish exhibit inherent barriers in membrane permeability to conventional transfection reagents, and the limited purity of germ stem cells in primary cultures may contribute to reduced transfection efficiency [48,49]. Current evidence indicates that transfection efficiency in primary cultures of freshwater fish germ stem cells rarely exceeds 5% in zebrafish PGCs [50], which is consistent with our observations in YRSSCs. Although adenoviral vectors demonstrate improved transduction efficiency, their infectivity remains constrained by species-specific variations in cell surface receptor distribution and internalization pathways [51]. Future optimization may involve species-specific modification of viral capsid proteins or the development of novel approaches combining electroporation with nanoparticle-based delivery systems [52]. These preliminary data provide valuable methodological references and establish baseline parameters for subsequent genetic manipulation studies in fish germ cells.

### 4.3. In Vitro Differentiation of YRSSCs

For most aquacultured fish species, producing functional sperm from spermatogonial cells cultured in vitro remains a challenge. In vitro culture systems provide opportunities to explore genetic and environmental factors affecting germ cell development and the regulatory mechanisms of gametogenesis. After suspending SSCs from red-flagged pufferfish in a medium containing 10 μM retinoic acid for 7 days, fibroblasts and astrocytes were observed, indicating the pluripotency of SSCs in vitro [53]. Co-culturing SSCs of hairtail with gonadal somatic cells, spheroid sperm cells were obtained after 5 days of induction, and cells with elongated tails forming sperm cells were observed after 7 days, and meiotic products with elongated tails were produced after 12 days [8]. In grass carp, spermatogonial cells prior to meiosis were isolated, and viable sperm were differentiated through an in vitro induction culture method [17]. In this study, YRSSCs induced by retinoic acid were able to produce epithelial cells, neurons, astrocytes, embryoid bodies, epithelial cells, spermatid-like cells, and sperm-like cells, indicating that the obtained SSCs possess pluripotency for in vitro differentiation.

Germ cell transplantation can be used to assess the functionality of fish SSCs. Transplantation of Nile tilapia SSCs, which were positive for PKH26, into busulfan-treated tilapia testes revealed that the transplanted SSCs could engraft, proliferate, and produce functional sperm within the tilapia testes, demonstrating the SSCs’ viability, mitotic activity, and self-renewal capacity [54]. Germ cell transplantation techniques, combined with cell cryopreservation, can protect endangered species by transplanting donor PGCs, germ stem cells, etc., into recipients with strong reproductive capabilities, ease of manipulation, and no endogenous germ cells, producing donor-derived sperm and eggs, thereby achieving the goals of preserving fish resources and protecting endangered species [55]. In this study, YRSSCs were transplanted into blastula-stage zebrafish embryos using microinjection techniques, and cell engraftment and proliferation were observed, indicating their pluripotency for in vivo differentiation.

The genetic improvement of most edible fish faces the challenge of long generations (1–4 years for common carp). However, the use of cryopreserved germ cells for artificial insemination, combined with CRISPR/Cas9 gene editing and cell transplantation techniques, can overcome the existing limitations of fish genetic improvement. CRISPR/Cas9, as a rapid, precise, and controllable gene engineering technology [56,57], can achieve targeted phenotypic modifications in organisms by delivering plasmid-based CRISPR/Cas9 delivery systems, in vitro transcribed sgRNA and Cas9 systems, or ribonucleoprotein (RNP)-based systems into recipient embryos through microinjection, electroporation, and other techniques [58]. This approach has been applied in various fish species, where silencing dnd in Atlantic salmon (Salmo salar) F0 mutants resulted in a phenotype lacking germ cells [59]; knockout of amhy in tilapia led to sex reversal [60]; knockout of myostatin (mstn) in carp, sea bream, and spotted gar promoted growth and muscle development [61,62,63]; and knockout of disease resistance-related genes created model animals lacking target genes [64]. Liu delivered CRISPR/Cas9 ribonucleoproteins into medaka cells via electroporation, achieving a knockout mutation rate of up to 50% in haploid cells and a maximum mutation efficiency of 61.5% in diploid cells [65]. Subsequently, Pan induced mutations in the tyr gene of medaka SG3 by injecting Cas9 mRNA and a CMV promoter-driven SpCas9/tRNA-gRNA system into SG3 [66]. Utilizing the promising gene knockout technology for targeted gene knockout in carp germ stem cells can shorten the breeding cycle of new carp varieties, accelerate the creation of new breeds, and promote the development of the aquaculture industry.

## 5. Conclusions

In this study, we established a method for the culture and cryopreservation of carp SSCs and obtained a stable SSC line from the Yellow River common carp (YRSSCs), which exhibited characteristics of germ stem cells, including strong positive alkaline phosphatase staining and positive expression of germ cell-specific genes (*vasa*, *nanos1*, *nanos3, klf4*-*2*). Additionally, YRSSCs demonstrated stable proliferation and differentiation capabilities in vitro, which could be induced to differentiate into neural cells, astrocytes, spermatid-like cells, and sperm-like cells. In vivo, YRSSCs exhibited self-renewal and differentiation ability, successfully colonizing the gonadal ridge of zebrafish after transplantation into embryos. The establishment of YRSSCs facilitates the exploration of the differentiation potential and regulatory mechanisms of fish GSCs, laying the groundwork for further research on fish germ cell transplantation and providing new avenues for fish breeding.

## Data Availability

Data is contained within the article.

## Supplementary Materials

The following supporting information can be downloaded at: https://www.mdpi.com/article/10.3390/biology14050536/s1. Figure S1: Results of RT-PCR agarose gel electrophoresis of vasa gene; Figure S2: Results of RT-PCR agarose gel electrophoresis of foxl2a and β-actin gene; Figure S3: Results of RT-PCR agarose gel electrophoresis of nanos1 gene; Figure S4: Results of RT-PCR agarose gel electrophoresis of *nanos3* gene; Figure S5: Results of RT-PCR agarose gel electrophoresis of klf4-2 gene; Figure S6: Utilizing RNA-FISH technology, the genes vasa-a and vasa-b are co-localized within YRSSCs; Table S1: Specific primers for male and female identification of Yellow River carp; Table S2: RT-PCR Primer Sequence; Table S3: RNA-FISH target gene probe sequences; Table S4: RNA-FISH Hairpin Probe Sequence.

## Abbreviations

The following abbreviations are used in this manuscript:

SSCs	spermatogonial stem cells
RNP	ribonucleoprotein
PGCs	primordial germ cells
