# Supplementary figures and images for: Establishment and Characteristics of the Spermatogonial Stem Cell Line from the Yellow River Carp (Cyprinus carpio haematopterus)
Source: Biology (Basel). 2025 May 12;14(5):536. doi: 10.3390/biology14050536 (PMC12109047; doi:10.3390/biology14050536)

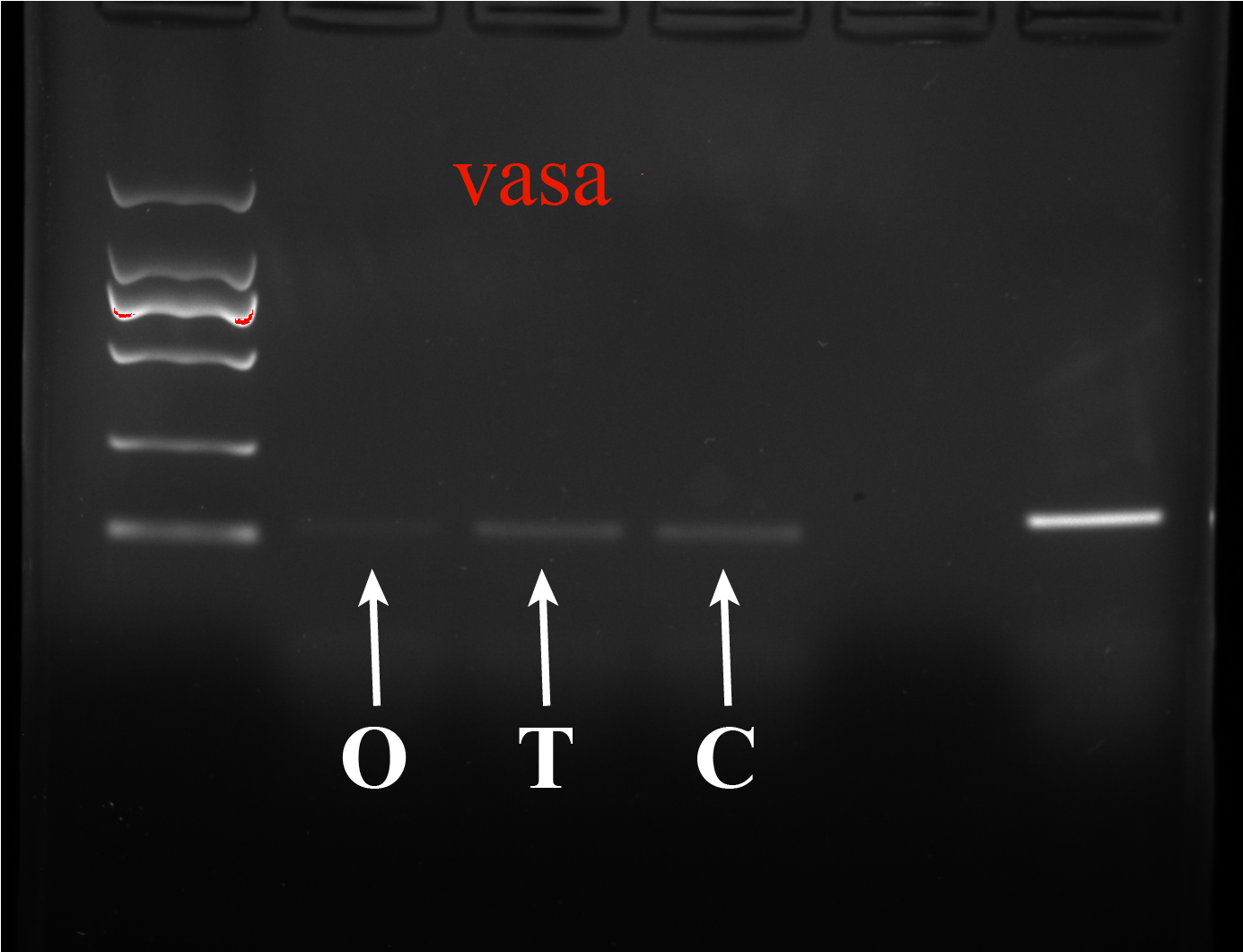

Supplement: Supplementary file 1 [file biology-14-00536-s001.zip › Supplementary Materials/Figures/Figure S1.png]

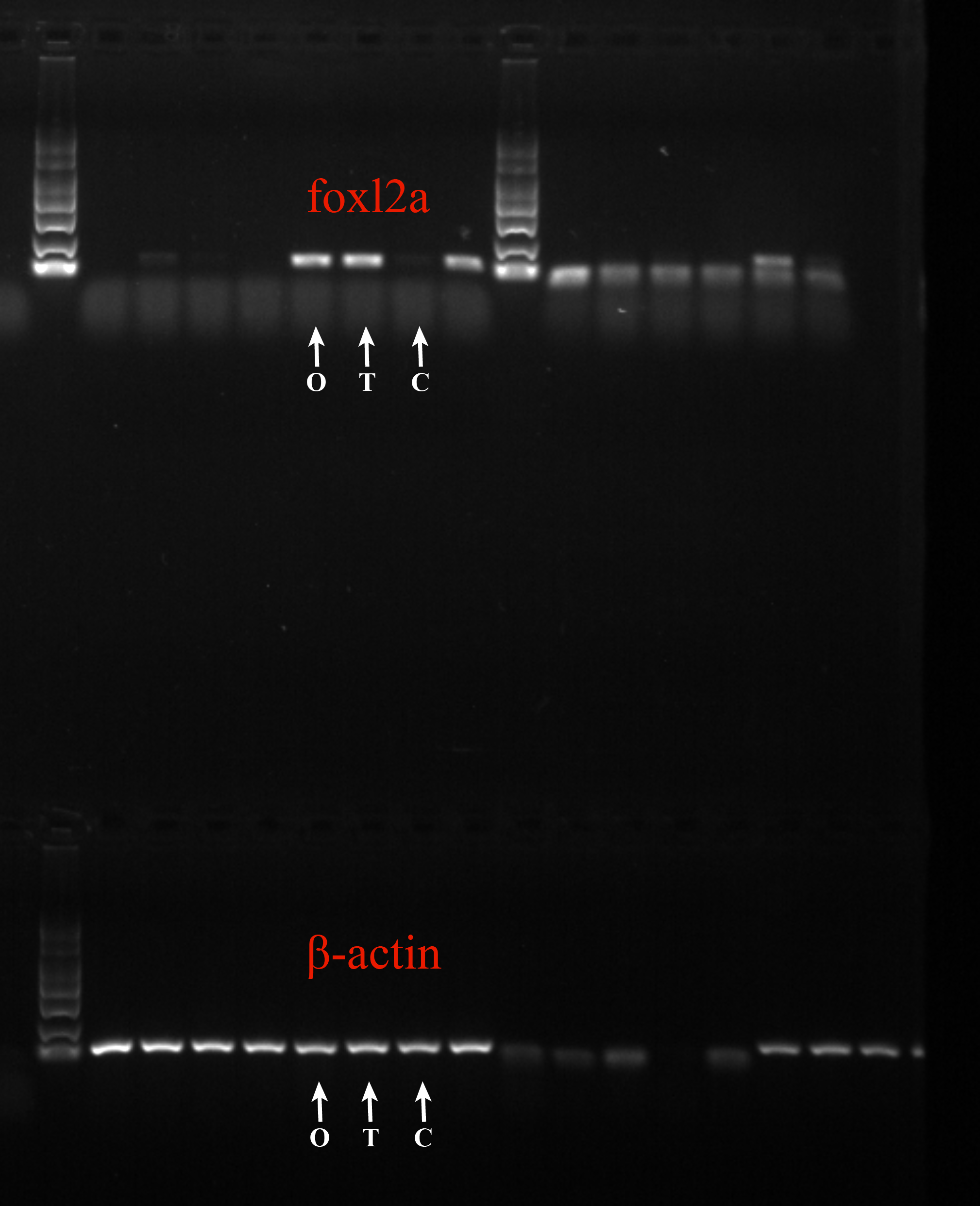

Supplement: Supplementary file 1 [file biology-14-00536-s001.zip › Supplementary Materials/Figures/figure S2.png]

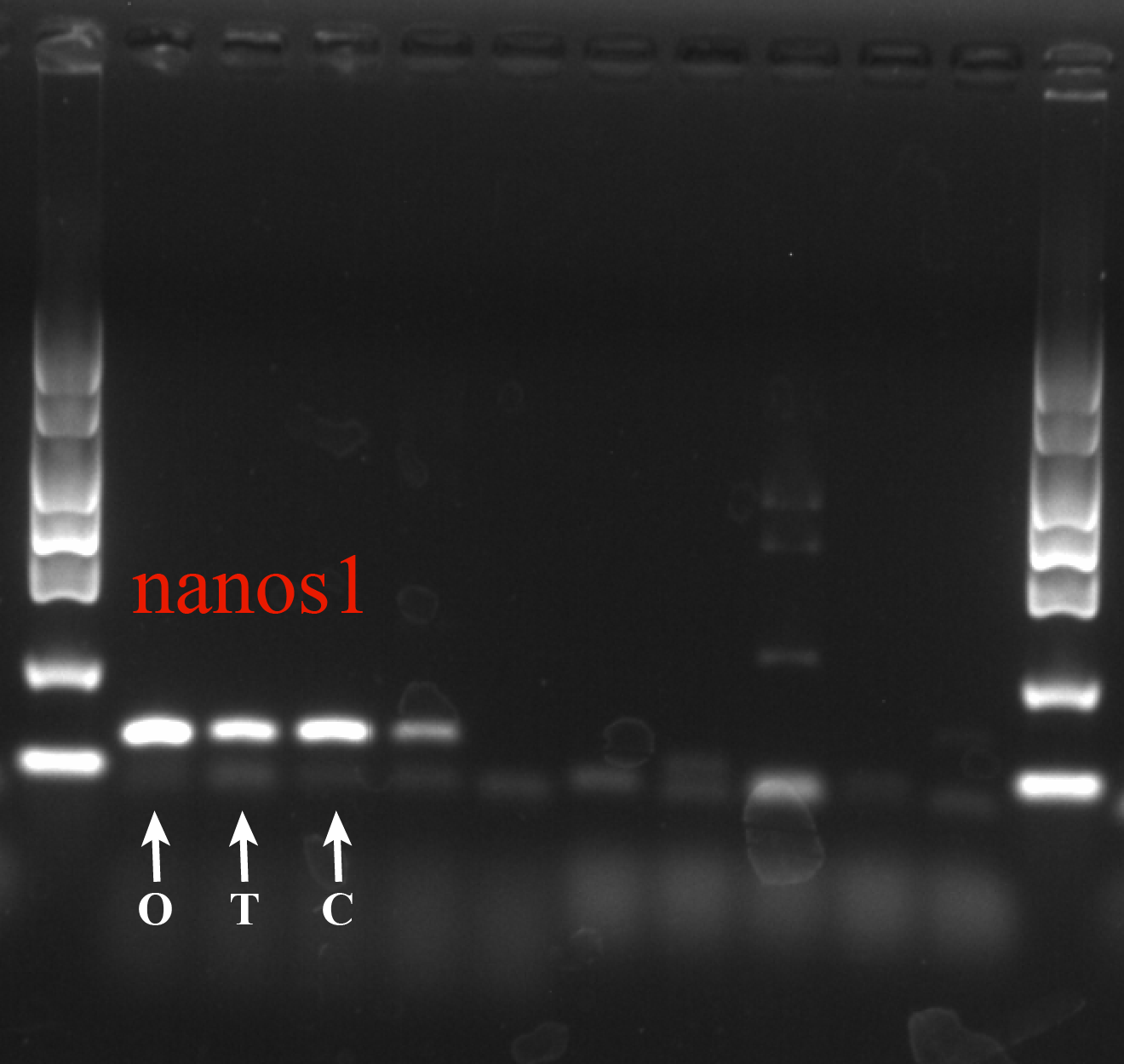

Supplement: Supplementary file 1 [file biology-14-00536-s001.zip › Supplementary Materials/Figures/Figure S3.png]

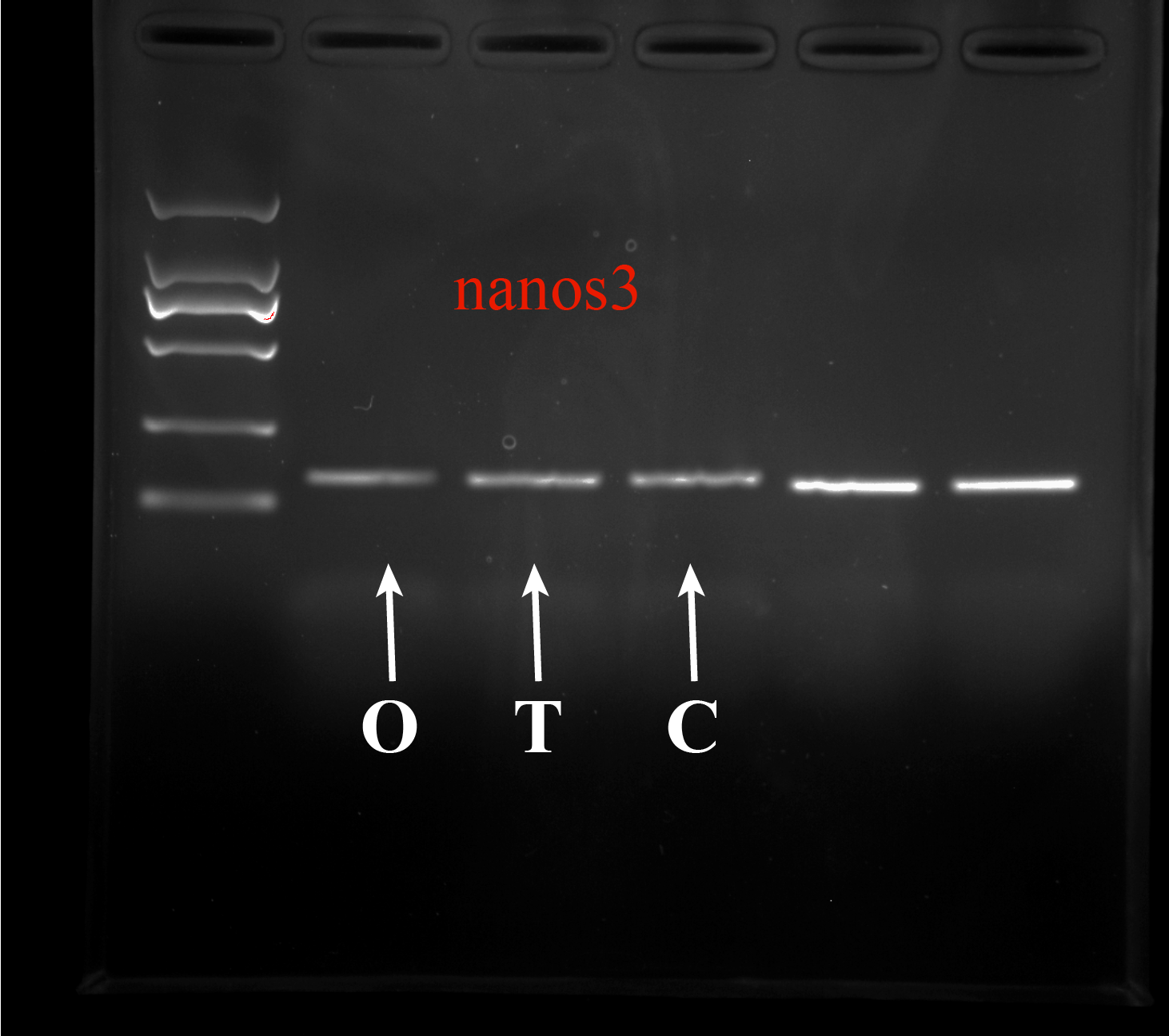

Supplement: Supplementary file 1 [file biology-14-00536-s001.zip › Supplementary Materials/Figures/Figure S4.png]

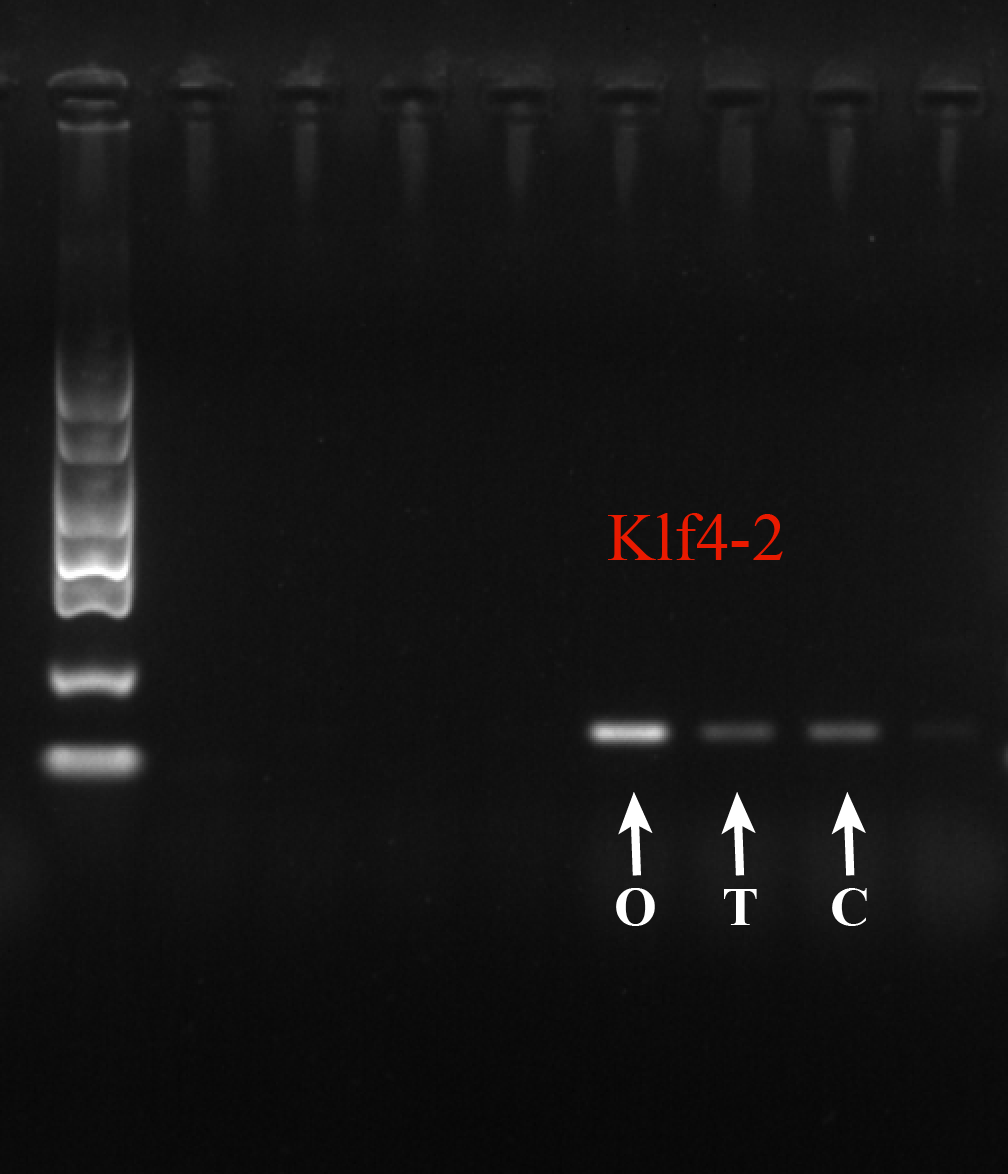

Supplement: Supplementary file 1 [file biology-14-00536-s001.zip › Supplementary Materials/Figures/Figure S5.png]

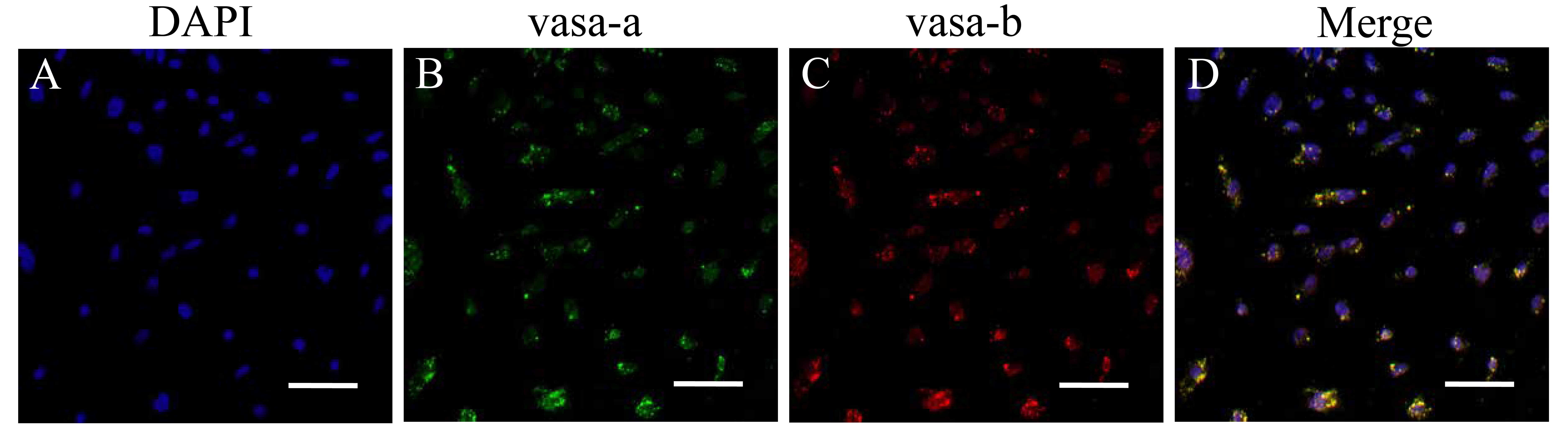

Supplement: Supplementary file 1 [file biology-14-00536-s001.zip › Supplementary Materials/Figures/figure S6.png]
